# Short‐ And medium‐term outcomes of reduced‐port laparoscopic surgery in elderly patients with upper rectal cancer: A retrospective cohort study

**DOI:** 10.1002/cam4.3070

**Published:** 2020-06-03

**Authors:** Huawen Wu, Zhijian Zheng, Lewei Xu, Yingying Wu, Ziyi Guan, Wenhuan Li, Guofu Chen

**Affiliations:** ^1^ Department of Surgery Wenling First People's Hospital Wenling City People’s Republic of China

**Keywords:** elderly, gastrointestinal surgery, laparoscopy, prognosis, rectal cancer

## Abstract

**Background:**

To investigate the short‐ and medium‐term outcomes of using a reduced‐port laparoscopic surgery (RPLS), compared to multi‐port laparoscopic surgery (MPLS), for the treatment of upper rectal cancer (URC) among elderly patients.

**Methods:**

We conducted a retrospective analysis of the clinical and follow‐up data of 181 elderly patients with URC, who underwent radical laparoscopic surgery at our hospital, between January 2015 and January 2019. Among these 181 cases, 62 underwent RPLS and 119 MPLS.

**Results:**

Compared to MPLS, RPLS decreased the length of surgical incision, lower pain on postoperative days 1 and 2, decreased the time to first flatus after surgery, as well as the time to mobilization after surgery. There was no difference between the short‐term outcomes between the two laparoscopic approaches, and no difference in the 3‐year disease‐free and overall survival rate.

**Conclusion:**

Compared to MPLS, RPLS provides several advantages for the treatment of URC among elderly individuals, including a shorter length of surgical incision, reduced postoperative pain, shorter time to first flatus after surgery, earlier mobilization, and better cosmetic outcomes. These advantages are achieved with no difference in the length of surgery, nor in the 3‐year disease‐free and overall survival rate, compared to MPLS.

## Graphical abstract

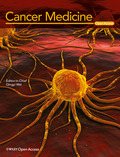

## 1 INTRODUCTION

Since the early 1990s, laparoscopic surgery (LS) has been widely used for the treatment of colorectal cancer, providing better cosmesis, faster postoperative recovery, shorter hospital stay, and lower incidence of perioperative complications, compared to open surgery, while having comparable safety and efficacy in oncology treatment.^1^, ^2^, ^3^, ^4^, ^5^ With the advancement of minimally invasive surgery, surgical oncologists have been exploring methods that require smaller incisions and a shorter recovery time. In 2008, Bucher et al reported on the application of single‐port laparoscopic surgery (SPLS) for the treatment of colorectal cancer.^6^ SPLS uses a small incision around the patient's navel, with insertion of instruments into the abdominal cavity via a multichannel access port.^6^ SPLS provides several advantages over a multi‐port laparoscopic (MPLS) approach, including: smaller incisions; reduced postoperative pain; shorter length of hospital stay; faster recovery; and little‐to‐no scarring.^7^ However, SPLS does present technical disadvantages compared to MPLS or an open surgical approach, including difficulty in obtaining a sufficient surgical field exposure, as well as interference among instruments and the requirement for greater surgical expertise.^7^ For these reasons, the indications for SPLS are currently limited to relatively simple surgeries, such as cholecystectomy and appendicectomy.

For radical treatment of colorectal cancer, as a reduced‐port laparoscopic surgery (RPLS), SPLS would present additional specific challenges.^8^, ^9^, ^10^, ^11^, ^12^, ^13^, ^14^, ^15^, ^16^, ^17^, ^18^, ^19^, ^20^, ^21^, ^22^, ^23^ First, the duration of surgery is significantly longer than for traditional laparoscopic surgery. The longer duration of anesthesia required may lead to postoperative circulatory, respiratory, and hepatorenal disturbances, which increase the incidence of perioperative complications. Second, SPLS may fail to achieve radical treatment of colorectal cancer due to the difficulty in achieving complete dissection of the lymph nodes adjacent to the inferior mesenteric arterial and venous roots, which would increase the risk for postoperative tumor recurrence. Third, difficulty in achieving sufficient tissue tension may make it difficult to clearly observe the abdominal anatomy, which would increase the risk of damage to nerves, blood vessels, and the mesenterium.^8^, ^9^, ^10^, ^11^, ^12^, ^13^, ^14^, ^15^, ^16^, ^17^, ^18^, ^19^, ^20^, ^21^, ^22^, ^23^ Our study addresses these limitations by comparing the application of RPLS, which includes SPLS, to MPLS for the treatment of upper rectal cancer (URC) among elderly individuals.

## 2 PATIENTS AND METHODS

This study complied with the Declaration of Helsinki. This retrospective research was approved by the ethics review board of our institution. The need for informed consent from all patients was waived because of retrospective study, not prospective trial.

Eligible patients for our retrospective study were the 181 elderly patients (age ≥ 65 years) who underwent laparoscopic surgery for URC at our hospital, between January 2016 and January 2019. URC was defined as lower margin of tumor between 10 and 15 cm from the anal verge by rigid sigmoidoscopy. These patients were screened on the following inclusion criteria: confirmed diagnosis of colorectal cancer by preoperative pathological studies; a clinical tumor stage T1‐3N0‐2M0; no prior history of malignant tumors; no indications for emergency surgery, such as bowel obstruction, intestinal perforation, and peritonitis; and no dysfunction of important organs, such as the heart and lungs. After screening, 181 patients were included in our study group, 62 cases treated using RPLS and 119 using MPLS. All surgeries were carried out by the corresponding author. Before this study, he has successfully completed 50 RPLS.

## 3 SURGICAL METHODS

### 3.1 RPLS

A multichannel trocar was inserted periumbilically to serve as the laparoscopic observation port and auxiliary surgical port; the auxiliary surgical clamp was inserted through this port. Another 10‐mm trocar was inserted via McBurney's point into the lower right abdominal quadrant as the main surgical port, with the ultrasound knife inserted through this port. The abdominal cavity was first explored for the presence of metastases on the surface of the liver and malignant ascites. An incision was then made at the retroperitoneum, 10 cm below the bifurcation of the abdominal aorta, in order to access Toldt's fascia. Dissociation of the fascia was performed under gas insufflation to maintain the pneumoperitoneum. After dissociating the fascia in cephalic and lateral directions, a cut was made at the level of the inferior mesenteric artery (IMA). After further dissociation of the fascia laterally, the inferior mesenteric vein (IMV) was ligated on the same plane as the IMA. Dissociation of the fascia was then further extended laterally and downwards to reach the left peritoneum. Caution was taken to preserve the nerves and ureters, located below the peritoneum. Next, the peritoneum to the left of the sigmoid colon was opened from the lateral side, and dissociation was continued medially until the presacral space was reached. At this point, the mesorectum was dissociated in a downward direction, extending beyond the lower border of the tumor to reach the site for rectal excision and anastomosis. At this point, gas insufflation was switched off.

The rectum and rectal mesentery were then resected and removed through the wound using a protractor/retractor. The intestinal canal was transected at a distance of about 10 cm from the tumor. The incision used for the protractor/retractor was closed and the pneumoperitoneum was reestablished. A stapler was then inserted via the anus, and the anastomosis was completed under direct observation. After hemostasis was achieved, the gauze was removed from the peritoneal cavity and the wound surface rinsed. A pelvic drainage tube was inserted and adequately fixed, and the surgical wound was then closed layer by layer.

### 3.2 MPLS

MPLS was performed using a 5‐port approach, including the following: a 10‐mm periumbilical observation port; a 10‐mm main surgical port inserted into the lower abdominal quadrant via McBurney's point; a 5‐mm incision at the intersection point between the midline of the right clavicle and the umbilical line, used for the surgeon's left‐hand operation port; and a 5‐mm incision at the intersection point between a line connecting the midpoint of the left clavicle and the umbilical line and a line connecting the midpoint of the left clavicle and the anterior superior iliac spine. The other surgical procedures were the same as for RPLS.

Dindo‐Clavien classification was used to classify postoperative 30‐day complications. Grade III or higher grade complications were considered as major.^24^ If the patient suffered more than two complications, only the highest one would be considered in the data analysis. Death within 30 days of laparoscopic surgery was considered as perioperative mortality. Indications for adjuvant chemotherapy are tumors with a pathological high‐risk stage II or stage III, and patients without contraindications to adjuvant chemotherapy. The specific chemotherapy regimens were determined by medical oncologists.

The final follow‐up was conducted in September 2019. Patients were regularly followed in the outpatient department every 3 months for the first postoperative year, every 4 months for the next 2 years, and then annually until the death of the patient.

Data were calculated as means and standard deviations for variables following normal distribution and were analyzed using *t* tests. For data not normally distributed, results were expressed as medians and ranges and compared using nonparametric tests. Differences in semiquantitative results were analyzed using the Mann‐Whitney *U*‐test. Differences in qualitative results were analyzed using the Chi‐square test or Fisher's exact test, as appropriate. *P* < .05 was considered to indicate statistical significance. The Statistical Package for the Social Sciences (SPSS) 13.0 (SPSS Inc) was applied.

## 4 RESULTS

There were no significant differences in the baseline characteristics of patients in the RPLS and MPLS groups (Table 1).

**Table 1:** Baseline characteristics of the two groups

	RPLS (n = 62)	MPLS (n = 119)	*P* value
Age (y)	67 (65‐75)	66 (66‐77)	.557
Sex			
Male	41	76	
Female	21	43	
BMI (kg/m^2^)	22 (19‐25)	21 (18‐27)	.247
ASA score			.804
I	39	77	
II	19	35	
III	4	7	
Clinical stage (cTNM)			.616
I	33	68	
II	22	39	
III	7	12	
Charlson comorbidity index (CCI)			.741
CCI ≤ 3	57	111	
CCI > 3	5	8	

Abbreviations: ASA, American Society of Anesthesiology; BMI, body mass index; CCI, Charlson comorbidity index; MPLS, multi‐port laparoscopic surgery; RPLS, reduced‐port laparoscopic surgery.

The following short‐term outcomes were also not significantly different between the two groups: operative time; intraoperative volume of blood loss; and the incidence and severity of complications within 30 days after surgery (Table 2). None of the patients, in either group, required a blood transfusion intraoperatively or within 30 days after surgery (Table 2). However, compared to the MPLS, RPLS was associated with a lower total length of surgical incision and lower pain scores on postoperative days 1 and 2, and a shorter time to first passage of flatus after surgery (Table 2). There were no pathological outcomes of the two groups (Table 3).

**Table 2:** Perioperative and postoperative outcomes of the two groups

	RPLS (n = 62)	MPL(n = 119)	*P* value
Operative time (min)	210 (160‐250)	200 (150‐240)	.147
Blood loss (ml)	160 (140‐240)	150 (110‐250)	.247
Conversion to multi‐port surgery	2	—	—
Conversion to open surgery	0	0	—
Blood transfusion	0	0	—
Time to pass first flatus (h, median and range)			.041
Postoperative pain score	1.5 (1‐3)	2 (1‐4)	
24 h, median (range)	4 (1‐5)	5 (2‐7)	.038
48 h, median (range)	3 (1‐5)	4 (1‐7)	.040
72 h, median (range)	1 (0‐3)	2 (1‐6)	.077
96 h, median (range)	1 (0‐3)	1 (0‐4)	.254
Postoperative hospital stay (d)	7 (5‐15)	8 (5‐19)	.179
Postoperative complications	8	14	.824
Anastomotic leakage	3	5	
Abdominal abscess	0	2	
Bowel obstruction	2	3	
Pneumonia	1	3	
Urinary retention	1	2	
Arrhythmia	1	1	
Major complications	0	1	
Minor complications	7	13	
Postoperative 30‐d death	0	0	—
Length of minilaparotomy (cm)	4.5 ± 1.0	4.7 ± 1.1	.774
Length of total incision (cm)	7.4 ± 1.4	5.4 ± 1.2	.000

Note

Postoperative pain was measured by visual analogue scale (VAS) at 24, 48, 72, and 96 h after laparoscopy.

Abbreviations: MPLS, multi‐port laparoscopic surgery; RPLS, reduced‐port laparoscopic surgery.

**Table 3:** Pathological data of the two groups

	RPLS (n = 62)	MPLS (n = 119)	*P* value
Pathological stage (pTNM)			.537
I	29	54	.935
II	21	46	
III	12	19	
Lymph nodes resected	19 (14‐28)	21 (15‐31)	.128
Circumferential resection margin			.638
Positive (≤1 mm)	1	1	
Negative (>1 mm)	61	118	
Histologic differentiation			.423
Well	17	39	
Moderately	32	59	
Poorly	13	21	
Residual tumor (R0/R1/R2)	62/0/0	119/0/0	1.000

Abbreviations: MPLS, multi‐port laparoscopic surgery; RPLS, reduced‐port laparoscopic surgery.

**Figure 1:**
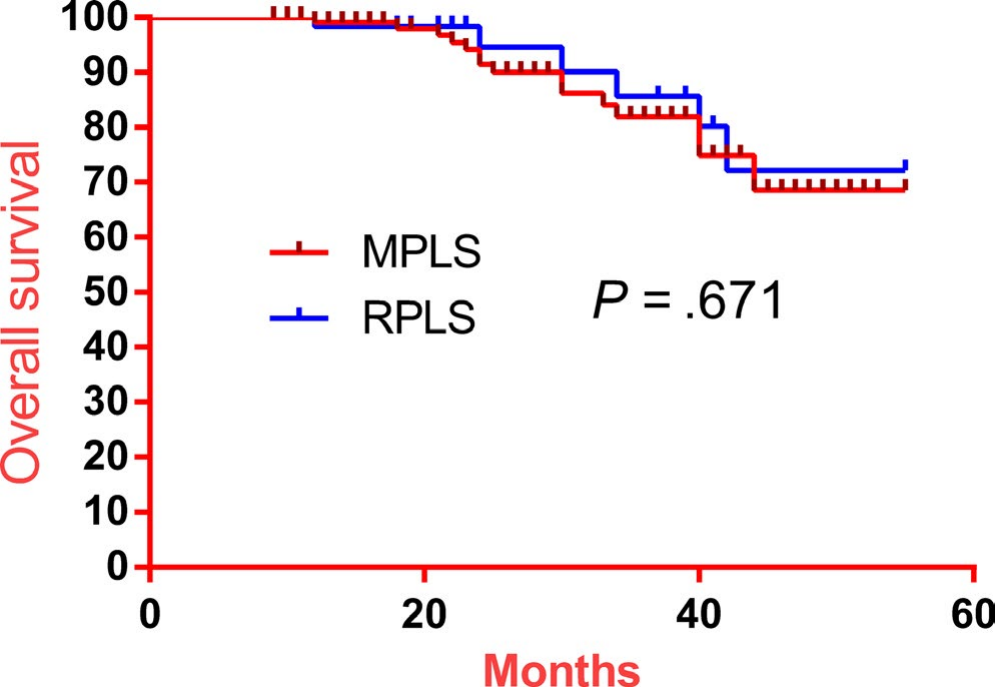
Comparison of overall survival rate between RPLS and MPLS groups. There was no significant difference between the two groups (*P* = .671)

**Figure 2:**
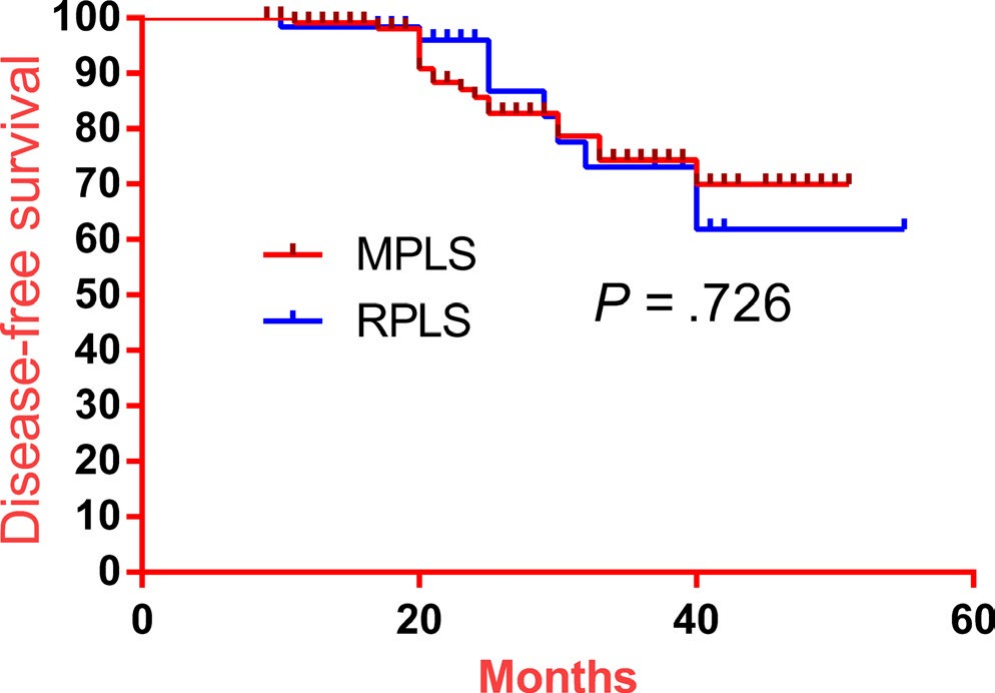
Comparison of disease‐free survival rate between the RPLS and MPLS groups. There was no significant difference between the two groups (*P* = .726)

All the patients complete the whole follow‐up. Over the follow‐up period, six deaths, due to tumor recurrence, were identified in the RPLS group, with four of these cases being localized recurrence and two metastatic tumors (Table 4). The 3‐year disease‐free survival rate and the 3‐year overall survival rate were not different between the two groups (Figures 1 and 2).

**Table 4:** The follow‐up data

	RPLS (n = 62)	MPLS (n = 119)	*P* value
Tumor recurrence during follow‐up	9	19	.798
Locoregional alone	3	7	
Distant alone	4	5	
Both locoregional and distant	2	7	
Port site	0	0	
Time to first recurrence (month, median, and range)	22 (10‐40)	19 (11‐42)	.457
Mortality during follow‐up	6	15	.559
Died of cancer recurrence	6	15	
Died of non‐oncological causes	0	0	

Abbreviations: MPLS, multi‐port laparoscopic surgeryRPLS, reduced‐port laparoscopic surgery.

## 5 DISCUSSION

Currently, minimally invasive surgical techniques, such as natural orifice transluminal endoscopic surgery and SPLS, have previously been used for colorectal surgery.^25^, ^26^, ^27^, ^28^, ^29^, ^30^, ^31^ Previous comparative study did not reveal any significant difference between the SPLS and MPLS groups with respect to length of hospital stay, rate of complications, number of lymph node resection, rate of positive margin, and rate of long‐term survival. SPLS, however, did provide an advantage over MPLS with regard to postoperative pain and total length of surgical incision. However, the longer operative time for SPLS and, most importantly, difficulty in achieving radical URC resection, compared to MPLS, have limited the indications of SPLS as a standard procedure for URC surgery.^7^ The challenge remains the need to achieve radical tumor resection while maintaining a balance between minimal invasiveness and an acceptable operative duration.^7^

RPLS builds on the basis of minimal invasiveness surgery, reducing interference between the main operation devices, enabling traction and counter‐traction between the two hands of the surgeon. Provision of a suitable angle between the main operation devices greatly reduces the technical difficulty of the surgery, particularly in the pelvic and rectal areas. These benefits could support RPLS as a general laparoscopic surgery in the near future, including for the treatment of URC.^9^, ^10^, ^11^, ^12^

Considering the general increase in life expectancy and improved health care, it is anticipated that the prevalence of colorectal cancer among elderly individuals will rise.^32^, ^33^ Several studies have reported on the benefits of MPLS over an open surgery for the treatment of geriatric colorectal cancer, providing a safe and effective treatment for URC among elderly individuals.^25^, ^26^, ^27^, ^28^, ^29^ Specifically, the short‐term clinical outcomes were better for MPLS than open surgery, with no difference in the long‐term clinical outcomes between the two surgical approaches. However, a thorough literature search did not identify research regarding the application of RPLS for the treatment of colorectal cancer among elderly individuals.

Elderly patients have a lower organ functional reserve and, thus, may be less tolerant to surgery than younger patients.^32^, ^33^ As such, the rate of postoperative complication is one of the key factors used in assessing the suitability of using RPLS for the treatment of URC among elderly patients. RPLS has a similar incidence rate of postoperative complications, postoperative recovery, and length of postoperative hospital stay as MPLS and traditional laparoscopic approaches.^8^, ^9^, ^10^, ^11^, ^12^, ^13^, ^14^, ^15^, ^16^, ^17^, ^18^, ^19^, ^20^, ^21^, ^22^, ^23^ Song et al compared the short‐term safety profile of RPLS and MPLS for colorectal surgery, reporting a decrease in postoperative pain and intraoperative volume of blood loss, as well as a shorter time to the first passage of flatus after surgery in patients who underwent RPLS compared to those who underwent MPLS.^13^ Zhang et al further reported an increase in patient satisfaction compared to MPLS.^34^ Our findings are in agreement with those of previous studies, providing evidence that as a minimally invasive technique, RPLS is associated with less postoperative pain and faster restoration of postoperative bowel function.

In conventional laparoscopic colorectal resection, five trocars are generally placed. Theoretically, reducing the number of trocars used would increase the technical difficulty of surgery, as well as prolonging the operative time. Of note on this issue, several studies have reported a comparable operative time for RPLS and MPLS,^9^, ^34^ while other studies reported a decrease in operative time with RPLS,^13^, ^16^ compared to MPLS. As RPLS uses one trocar, it requires less surgical assistance that the use of multiple trocars with MPLS. We must also consider that during MPLS procedures, assistance is often provided by trainees, with less clinical experience; the limited surgical experience of trainees with laparoscopic procedures could significantly prolong operative time. In this way, the reduction in the number of trocars for RPLS would be a distinct advantage over MPLS.

The likelihood of achieving radical treatment has been the primary consideration in selecting the surgical method for treatment of URC, and other cancers. Previous studies ^8^, ^9^, ^10^, ^11^, ^12^, ^13^, ^14^, ^15^, ^16^, ^17^, ^18^, ^19^, ^20^, ^21^, ^22^, ^23^ have reported similar pathological and long‐term oncological outcomes for RPLS and MPLS. In our study, we reported comparable medium‐term outcomes between RPLS and MPLS for the treatment of geriatric URC, including the number of lymph node resection, rate of positive margin, rate of tumor recurrence, the 3‐year disease‐free survival rate, and the overall survival rate. The findings of advantages of RPLS with regard to postoperative pain, time to first passage of flatus after surgery, and shorter time to the first out‐of‐bed activity after surgery, compared to MPLS, with no difference in the 3‐year disease‐free survival rate and the 3‐year overall survival rate would be specifically important in China, where the prevalence of people over the age of 65 years has been predicted to increase, with the cutoff age of 65 years used by the World Health Organization to define elderly individuals.

The limitations of our study need to be acknowledged. First, patients with mid‐low rectal cancer (MLRC) were not included in our study group. This is important as the difficulty in surgical treatment is greater for patients with MLRC than URC. Moreover, patients with locally advanced MLRC require neoadjuvant chemoradiotherapy, which can lead to increased tissue adhesion, increasing the difficulty of the surgery intervention. Second, as our sample size was relatively small, multicenter, prospective, randomized control studies are needed to further confirm the benefits and safety of using RPLS for the treatment of URC among elderly individuals. Lastly, as RPLS was first performed at our hospital in January 2015, the follow‐up period of this study is relatively short; therefore, further studies are needed to follow‐up the 5‐year and 10‐year survival rates of patients.

## 6 CONCLUSION

RPLS provides several advantages for the treatment of URC among elderly individuals, including a shorter length of surgical incision, reduced postoperative pain, shorter time to first flatus after surgery, earlier mobilization, and better cosmetic outcomes. These advantages are achieved with no difference in the length of surgery, nor in the 3‐year tumor‐free and overall survival rate, compared to MPLS.

## CONFLICT OF INTEREST

None of the authors have any financial interest relevant to the work presented in this manuscript.

## CONFLICT OF INTEREST

None.

## AUTHOR CONTRIBUTION

Huawen Wu and Zhijian Zheng contributed to study conception and design, Lewei Xu and Yingying Wu contributed to acquisition of data, Ziyi Guan, Wenhuan Li, and Guofu Chen contributed to analysis and interpretation of data, Huawen Wu and Zhijian Zheng contributed to drafting of manuscript, and Huawen Wu and Zhijian Zheng contributed to critical revision.

## ETHICAL APPROVAL

This study complied with the Declaration of Helsinki. This retrospective research was approved by the ethics review board of our institution. The need for informed consent from all patients was waived because of retrospective study, not prospective trial.

## Data Availability

All data included in this study are available upon request by contacting with the corresponding author.
